# Gas adsorption meets deep learning: voxelizing the potential energy surface of metal-organic frameworks

**DOI:** 10.1038/s41598-023-50309-8

**Published:** 2024-01-26

**Authors:** Antonios P. Sarikas, Konstantinos Gkagkas, George E. Froudakis

**Affiliations:** 1 Department of Chemistry, University of Crete, Voutes Campus, 70013 Heraklion, Crete Greece; 2 Advanced Technology Division, Toyota Motor Europe NV/SA, Technical Center, Hoge Wei 33B, 1930 Zaventem, Belgium

**Keywords:** Computational methods, Cheminformatics

## Abstract

Intrinsic properties of metal-organic frameworks (MOFs), such as their ultra porosity and high surface area, deem them promising solutions for problems involving gas adsorption. Nevertheless, due to their combinatorial nature, a huge number of structures is feasible which renders cumbersome the selection of the best candidates with traditional techniques. Recently, machine learning approaches have emerged as efficient tools to deal with this challenge, by allowing researchers to rapidly screen large databases of MOFs via predictive models. The performance of the latter is tightly tied to the mathematical representation of a material, thus necessitating the use of informative descriptors. In this work, a generalized framework to predict gaseous adsorption properties is presented, using as one and only descriptor the capstone of chemical information: the potential energy surface (PES). In order to be machine understandable, the PES is voxelized and subsequently a 3D convolutional neural network (CNN) is exploited to process this 3D energy image. As a proof of concept, the proposed pipeline is applied on predicting ${\hbox {CO}_{2}}$ uptake in MOFs. The resulting model outperforms a conventional model built with geometric descriptors and requires two orders of magnitude less training data to reach a given level of performance. Moreover, the transferability of the approach to different host-guest systems is demonstrated, examining ${\hbox {CH}_4}$ uptake in COFs. The generic character of the proposed methodology, inherited from the PES, renders it applicable to fields other than reticular chemistry.

## Introduction

Reticular chemistry, *the science and art of combining molecular building blocks to form extended periodic structures*^1^, has endowed chemists and material scientists with a vast chemical space, the latter serving as a giant toolbox that can help them to solve a wide variety of problems. Metal-organic frameworks (MOFs), a class of nanoporous materials composed of metal ions/clusters and organic linkers^2^, exemplify this idea. Owing to their exceptionally high porosity and surface area^3^ along with their tunable nature have burgeoned as prominent materials for gas-adsorption related applications^4,5^. One such example, is carbon capture and storage^6^, where MOF-based sorbents are considered green and efficient solutions.

The inherent combinatorial character of MOFs has given birth to large databases of either in vitro^7,8^ or in silico synthesized materials^9–12^. Although a plethora of choices is desirable, an immense materials space inevitably complicates the efficient identification of the best candidates. The large size of current and prospective MOFs databases^13^, precludes approaches such as experimental synthesis and performance characterization, since a single laboratory study can range from weeks to months. Performance assessment based on molecular simulations^14^, significantly ameliorates the time penalty that accompanies the evaluation of a single structure. Nevertheless, the overwhelming number of MOFs that require filtering renders brute-force computational screening suboptimal.

The ever-increasing amount of data requires methods that are able to handle them efficiently and effectively. Machine learning (ML) techniques satisfy the aforementioned requirements and can accelerate the identification of promising materials by means of predictive models^15–24^. Given a mathematical description of a structure (input) and a property of interest (output), a supervised ML algorithm seeks to build a model for the underlying structure-property relationship. In ML parlance, inputs and outputs are known as *descriptors (or features)* and *labels*, respectively. *“Garbage in, garbage out”* applies, which entails that high-performing ML models are possible only if information-rich descriptors are employed.

With regards to gas adsorption in MOFs, a commonly used set of descriptors are the so-called *geometric* ones^25–27^ including properties like void fraction and gravimetric surface area. Although these descriptors lead to fruitful results when used to predict gas uptake at high pressures, they fall short as we transition to the low pressure regime, especially when modeling gases with non-negligible electrostatic interactions, e.g. ${\hbox {CO}_2}$ and ${\hbox {H}_2}$. The origin of these shortcomings is rooted to the inability of these descriptors to capture the fundamental factor that governs adsorption: *host-guest interactions*. Attempts have been performed to address the limitations of geometric descriptors, i.e. to capture the energetics of adsorption, giving rise to the so-called *energy-based* descriptors^28–30^.

**Figure 1:**
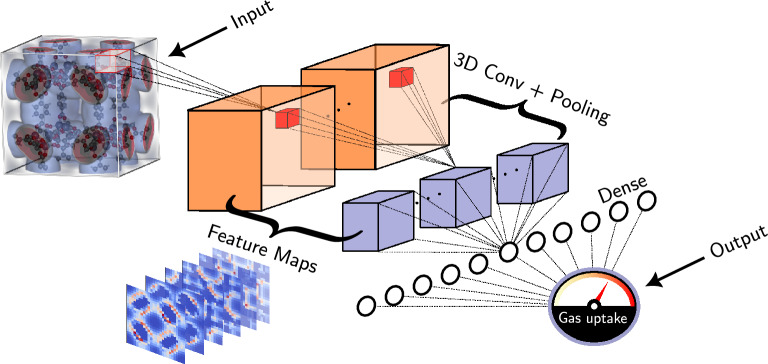
Generalized framework for predicting adsorption properties using the entire PES as descriptor. Starting from PES as raw input, a CNN extract its features, and utilizes them to predict a property of interest (hereon gas uptake). The iRASPA^31^ software was used for the visualization of IRMOF-1 PES and structure.

**Figure 2:**
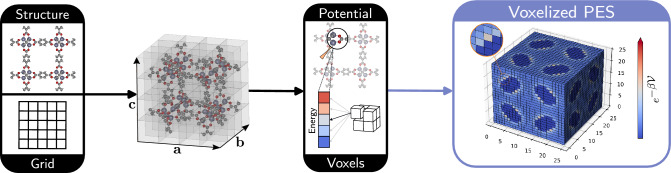
Workflow to construct the voxelized PES. The latter can then be processed by a CNN. The iRASPA^31^ software was used for the visualization of IRMOF-1 structure.

 One such work was performed by Bucior et al.^32^ where bins from the unit cell’s energy histogram were used as descriptors, leading to ML models of remarkable accuracy with regards to ${\hbox {H}_2}$ and ${\hbox {CH}_4}$ uptake. In another study^33^, a set of hypothetical probe atoms were used to fingerprint the energetic landscape of the unit cell by averaging the interaction between the probe and framework atoms. Augmenting the set of geometric descriptors with these average interactions significantly increased the performance of ML models regarding ${\hbox {CO}_2}$, ${\hbox {H}_2\hbox {S}}$, ${\hbox {H}_2}$ and ${\hbox {CH}_4}$ uptake. Since *gas adsorption essentially boils down to the potential energy surface (PES)*, a natural question that arises is *why not use the PES itself as descriptor?*

In this work, we propose a generalized framework for predicting gaseous adsorption properties, free of hand-crafted features, *using solely the PES as descriptor* (Fig. 1). Two steps are central to this approach:

(i) a machine understandable format of PES
(ii) an algorithm capable of handling effectively this format

For the first step, a *voxelized* representation of the PES is adopted. In essence, the input can be thought as a 3D image of the material, where each 3D pixel, i.e. *voxel*, is colorized by the values of the potential energy. For the second step, we resort to a *deep learning*^34,35^ solution, namely a 3D convolutional neural network (CNN). Similar to reticular chemistry, in deep learning—a subfield of ML—*simple computational units (neurons) are combined to form (neural) networks*, the latter being able to extract useful information from the raw input, that is to extract features in a completely data-driven way.

It should be added that our pipeline doesn’t impose extra computational burden compared to the afore-described studies^32,33^, since in both of them the features are extracted from the PES or an approximation thereof. The essential difference lies on the feature extraction step: *here the algorithm decides what matters for the task at hand by looking on the data, removing the need for manual feature extraction*.

**Figure 3:**
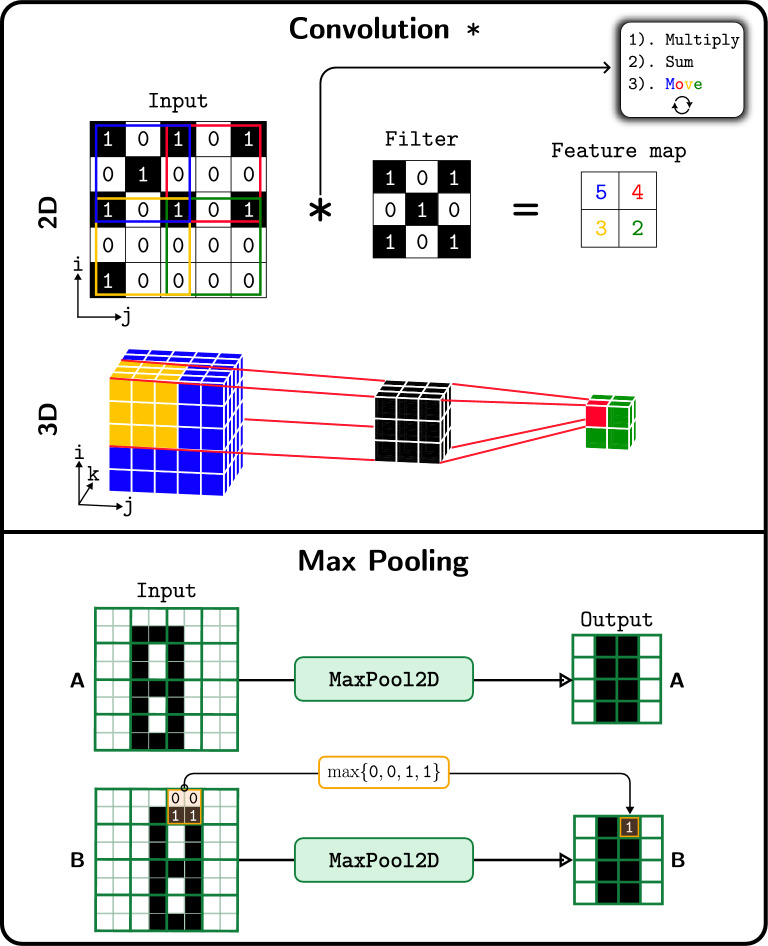
(Top) Convolution operation. (Bottom) Max pooling operation.

With respect to application of CNNs on MOFs, Cho et al.^36^ trained a CNN by representing the structure as a 3D binary matrix, with each matrix value indicating “available” or “non-available” adsorption sites in the structure. Notably, the CNN was capable of accurately predicting ${\hbox {CH}_4}$ adsorption isotherms of zeolites. Following up this work, in order to account for the chemical diversity of MOFs, Hung and co-workers^37^ trained a CNN on two 3D matrices that encode element and point-charge information. The CNN achieved remarkable accuracy on predicting Henry adsorption constants for ${\hbox {CH}_4}$ and ${\hbox {CO}_2}$ and improved compared to the binary matrix approach. Nevertheless, the fact that an *indirect representation of the PES* is used as a descriptor, entails that the information content the CNN receives is not maximal. *Maximum information content is possible only if the PES itself is used as a descriptor*. Here, the CNN is trained on a single 3D matrix instead of two but more importantly, *it looks directly on the PES*. In other words, we feed the CNN with the object that completely characterizes the sorption behavior of a material: the PES.

Since the PES uniquely combines the structural properties and the electronic structure of a material in real space, the proposed scheme is applicable in any host-guest system for predicting any adsorption property of interest. As a proof of concept, the suggested approach is applied on MOFs for predicting ${\hbox {CO}_2}$ uptake. The transferability of the approach is also demonstrated, examining ${\hbox {CH}_4}$ uptake on COFs. In both cases, the proposed pipeline is compared with conventional schemes where geometric descriptors are employed.

## Methods

### Voxelized PES

The steps for the calculation of the voxelized PES are schematically summarized in Fig. 2. As a first step, a 3D grid of size $n \times n \times n$ is overlayed over the unit cell of the material. Hereon, $n = 25$ as a balance between resolution and computational cost, since voxelization scales up as ${\mathcal {O}}(n^3)$. Next, each voxel centered at grid point ${\textbf{r}}_i$ is colorized with the interaction energy of a probe molecule at ${\textbf{r}}_i$ with the framework atoms.

**Figure 4:**
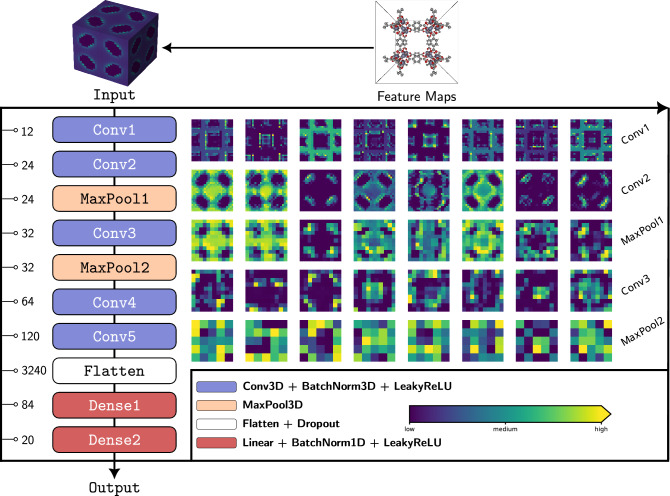
RetNet architecture and forward pass of IRMOF-1. For the sake of visualization, slices (feature maps are 3D matrices) of 8 feature maps from the first 5 layers are depicted. For Conv1 layer the 5th slice is depicted while for the other layers the 1st slice is depicted. All slices are collected along the 1st dimension of the corresponding 3D matrix. The iRASPA^31^ software was used for the visualization of IRMOF-1 structure.

In the proposed framework, the grid size and the type of the potential are treated as “hyperparameters” controlling the trade-off between information content and computational cost. *The ultimate representation of the PES* is achieved as $n \rightarrow \infty$ and when the voxels are filled with *energy values obtained from ab-initio calculations.* Because this study serves as a proof of concept, to facilitate the modelling of interactions, a spherical probe molecule is used and host-guest interactions are approximated with the Lennard-Jones (LJ) potential (for more details see Supplementary Information). To ease the calculation of energy voxels, the Python package MOX$\epsilon \lambda$ is introduced and used in all cases. For the remainder of this study, we use interchangeably the terms “voxelized PES” and “energy voxels”.

### Convolutional neural networks

CNNs are specialized neural network architectures to process image-like data^38–41^ based on *convolution* (Fig. 3). Convolving a filter with an image can be seen as *template matching*. When a local image patch matches the filter—template to be matched—the output is highly positive. Sliding the filter over the image and recording the output values produces a *feature map*. This notion generalizes to 3D. A *convolutional layer*, contains many *learnable filters*, each one of them looking for a different pattern and producing its own feature map based on feature maps from the previous layer or the raw image (in case of first convolutional layer). By composing many such layers, a CNN extract features hierarchically, with the level of (feature) abstraction increasing the deeper we go into the network.

Apart from convolutional layers, another common building block of CNNs are the *pooling layers*. The role of these layers is to *downsample (reduce the resolution) in a parameter-free way* the feature maps produced by convolutional layers. By downsampling in this manner, they reduce the memory-computational footprint of the CNN and also the number of parameters, thereby reducing the risk of overfitting^42^. A pooling layer takes as inputs the feature maps of the preceding convolutional layer and subsamples them by substituting the outputs in a small neighborhood of the feature map with a summary statistic^35^. Figure 3 illustrates the pooling layer used in this work, known as *max pooling*, which uses the $\max$ function to compute the summary statistic (same idea applies to 3D). From the same figure it can be also seen that small translations to the input (input B is just a shifted version of input A by 1 pixel to the right) produce the same output when passed through the max pooling layer, meaning that the latter introduces into the network some level of invariance to small translations^35,42^. The architecture of the 3D CNN used in this work called RetNet is schematically depicted in Fig. 4. More information regarding architecture and training details can be found on the Supplementary Information.

## Results

### Visualizing RetNet

A closer look at the “internals” of RetNet—the processing that energy voxels undergo as they pass through the network—trained on the MOFs dataset^9^, is provided in Fig. 4. For the sake of clarity, only some feature maps from the first five layers are visualized. It should be noted that each feature map of a given layer takes into account all the feature maps from the previous layer, the only exception being the pooling layers which just dowsample. For instance, each feature map of the Conv2 layer combines all the 12 feature maps from Conv1 layer whereas each feature map of the MaxPool1 layer is a downsampled version of the corresponding feature map in Conv2 layer. Although feature maps are not meant to be human interpretable (especially the ones found deeper in the network), it is worth to notice that the first two Conv layers highlight the texture of the structure. For example, it can be seen that the 3rd feature map from Conv1 layer outlines the skeleton of the framework.

The MaxPool2 layer is followed by two consecutive Conv layers and the Flatten layer flattens out all feature maps of Conv5 into a single vector (of size 3240) which is then processed by a fully connected neural network. Since Output is a linear layer (see Table S1 in the Supplementary Information), RetNet essentially does just the following:

1 $$\begin{aligned} \underbrace{\overbrace{{\textbf{x}}}^\text {PES}}_\text {input} \quad \longrightarrow \quad \underbrace{ \overbrace{\mathbf {\phi }({\textbf{x}};\mathbf {\theta })}^\text {fingerprint} }_\text {feature extraction} \quad \longrightarrow \quad \underbrace{ \overbrace{{\textbf{w}}^\top \mathbf {\phi }({\textbf{x}};\mathbf {\theta }) + w_0}^\text {gas uptake} }_\text {output} \end{aligned}$$

In other words, it extracts a fingerprint from the PES and then uses a linear model on top of this fingerprint to predict the gas uptake. All the layers between Input and Output layers (i.e. from Conv1 to Dense2) are responsible for this feature extraction step, with the size of the fingerprint being determined by the size of the Dense2 layer (a 20-dimensional vector, see also Figs. S6–S7 in the Supplementary Information). This *learnable fingerprint extraction step* (parameters $\mathbf {\theta }$ of $\mathbf {\phi }$ are learned during training), is what fundamentally differentiates our method compared to approaches that use *hand-crafted fingerprints*^32,33^. Feature extraction from the PES has been “unlocked” and is now part of the training phase.

**Figure 5:**
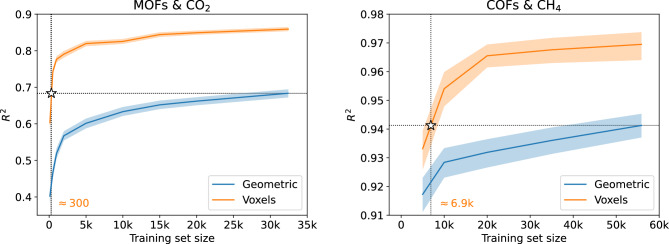
Performance on test set measured by $R^2$ (the higher the better) as function of the training set size for the models with geometric descriptors (RF) and energy voxels (CNN). Shaded areas correspond to the 95% CI. Coordinates of the white star denote the training set size (*x*-coordinate) where the CNN achieves the performance of the RF model (*y*-coordinate) trained with the maximum training set size. “Geometric” stands for geometric descriptors while “Voxels” stands for energy voxels.

### Learning curves

The learning curves for ML models built with energy voxels and geometric descriptors are depicted in Fig. 5. As can been seen, in the MOFs dataset, the CNN model ($R^2=0.859$) outperforms the Random Forest (RF) model ($R^2=0.690$) even with such a crude approximation for the PES (LJ potential doesn’t take into account electrostatic interactions). Notably, the CNN model requires two orders of magnitude less training samples, approximately 300, to reach the performance of the RF model. As stated previously, in this work we strived for minimal computational cost which means that still the information content of the voxelized PES is not maximized. As such, higher performance can be achieved by employing more refined potentials and *the upper limit with an ab-initio constructed PES*. Similar behavior is observed on the COFs dataset^43^. Again, the CNN model generalizes better, achieving a $R^2$ of 0.969 compared to 0.941 for the RF model. In this case, the CNN needs around one order of magnitude less training samples to match RF’s performance. The observation that in both cases the learning curve of the CNN model lies above the corresponding one of the RF model should be attributed (mainly) to the following two factors. First, the increased information content of the voxelized PES (input of CNN models) over the geometric descriptors (input of RF models). Second, the ability of CNNs to process image-like data (the voxelized PES is just a single-channel 3D image). It should be mentioned that another factor that gave a performance boost to our CNN models was the application of data augmentation during their training phase (see Figs. S2–S3 in the Supplementary Information).

## Discussion

We would like to point out that in the case of ${\hbox {CH}_4}$, which lacks dipole and quadrupole moment, the LJ potential approximates very well the true potential, which is reflected on the increased performance of the CNN compared to the ${\hbox {CO}_2}$ case. This observation along the fact that in both cases the same resolution was used, motivates focusing first on refining the potential than increasing resolution in order to maximize the information content of the voxelized PES and as such, the performance of the ML models^33^. For adsorbates like ${\hbox {CO}_2}$ and ${\hbox {H}_2}$, where electrostatic interactions with the framework atoms are non-negligible, an accurate representation of the voxelized PES necessitates the inclusion of these interactions. However, there is no free lunch and such refinements come at the price of assigning partial charges to each framework atom, which is a computationally intensive task. Fortunately, there are approaches^44–46^ that can assign partial charges extremely fast and with high fidelity via ML models, opening the door for efficient construction of an accurate voxelized PES.

Additionally, considering that the proposed framework bases its roots at interactions, which are ubiquitous in nature, renders it extremely modular and applicable to fields besides reticular chemistry. For example, if one is interested in predicting properties of organic molecules (e.g. solubility), a straightforward application of our framework is to voxelize the electrostatic potential map of the organic molecule and then use it as input to train a 3D CNN for predicting the property(ies) of interest.

Moreover, the fact that under the hood the proposed framework uses a member of the deep learning family, enables incorporation of transfer learning techniques^47,48^, which can greatly decrease the amount of reference data required for the CNN training. In transfer learning, the model leverages the knowledge it has gained by solving an original task to solve new but *similar to the original* tasks. For example, instead of retraining from scratch the CNN for every adsorption property of interest, one can train the CNN at a specific property (original task, e.g. gas uptake) and then fine-tune this pre-trained model to the other properties (new tasks, e.g. gas selectivity). A good pre-trained model will require less training data to perform well in the new task, because it can exploit the shareable knowledge it has acquired by solving the original task.

Since the performance of a ML model depends highly on the informativeness of the descriptor(s) and the algorithm of choice, refinements of the potential used to model host-guest interactions, architecture modifications along with inclusion of transfer learning techniques, can further improve the performance and data efficiency of the suggested pipeline. As a final note, it should be remembered that reticular chemistry and chemistry are three dimensional, and if we are to “machine-learn them” properly, we ought to respect it. We envision that our study will motivate the adoption of three dimensional inputs in future chemistry-oriented ML works.

### Supplementary Information

Supplementary Information.

## Data Availability

The energy voxels for MOFs are publicly available in: https://figshare.com/articles/dataset/RetNet/24598845. The energy voxels for COFs are available from the corresponding author upon request.

## Code availability

MOX$\epsilon \lambda$, is hosted on GitHub: https://github.com/frudakis-research-group/moxel and can be installed from PyPI: https://pypi.org/project/pymoxel/. Documentation for the package is available at: https://moxel.readthedocs.io/en/stable. A PyTorch implementation of RetNet is available on GitHub: https://github.com/frudakis-research-group/retnet.
