# Supplementary material for: Gas adsorption meets deep learning: voxelizing the potential energy surface of metal-organic frameworks
Source: Sci Rep. 2024 Jan 26;14:2242. doi: 10.1038/s41598-023-50309-8 (PMC10817925; doi:10.1038/s41598-023-50309-8)
Supplement: Supplementary file 1 — Supplementary Information. [file 41598_2023_50309_MOESM1_ESM.pdf]

Gas adsorption meets deep learning: voxelizing the potential energy surface of metal-organic frameworks

Antonios P. Sarikas[1], Konstantinos Gkagkas[2], and George E. Froudakis[1,*]

[1]Department of Chemistry, University of Crete, Voutes Campus, GR-70013 Heraklion, Crete, Greece
[2]Advanced Technology Division, Toyota Motor Europe NV/SA, Technical Center, Hoge Wei 33B, 1930 Zaventem, Belgium
[*]frudakis@uoc.gr

ABSTRACT

Not Applicable.

Voxelized PES

At each voxel centered at grid point $\mathbf{r}_i$, the interaction energy $V(\mathbf{r}_i)$ between the spherical probe molecule and framework atoms was calculated using the Lennard-Jones potential, as following:

$$V(\mathbf{r}_i) = \sum_{\substack{j=1 \\ r_{ij} \leq r_c}}^{N} 4\varepsilon_{ij} \left[\left(\frac{\sigma_{ij}}{r_{ij}} \right)^{12} - \left(\frac{\sigma_{ij}}{r_{ij}} \right)^{6} \right] \tag{1}$$

where N is the number of framework atoms, r_{ij} is the distance between the j-th framework atom and the probe molecule, r_c is the cutoff radius, which was set to 10 Å, and ε_{ij}, σ_{ij} combine the ε and σ value of the probe molecule and the j-th framework atom according to the Lorentz-Berthelot mixing rules:

$$\sigma_{ij} = \frac{\sigma_i + \sigma_j}{2} \qquad \text{and} \qquad \varepsilon_{ij} = \sqrt{\varepsilon_i \varepsilon_j} \tag{2}$$

For the probe molecule, $\varepsilon_i/k_B = 50\,K$ and $\sigma_i = 2.5\,Å$. For the framework atoms, the corresponding parameters from the Universal Force Field[1] were used.

Geometric overlap between a grid point, i.e. the position of the probe molecule, and a framework atom can lead to a very large (even infinite) energy value (highly repulsive interaction), which is detrimental for the training of a neural network. For example, if a single voxel of just one material takes the value of infinity, training can't even start since this infinite value leads to `NaNs` during standardization (see "Preprocessing and training details" subsection). In order to avoid such problems we fill each voxel with $e^{-\beta V(\mathbf{r}_i)}$, which tends to 0 as $V(\mathbf{r}_i) \to \infty$, where $\beta = \frac{1}{k_B T}$, k_B is the Boltzmann constant and T is the temperature, which was set at 298 K.

Datasets

Known for their "data hungriness", deep neural networks require relatively large amount of data to unleash their full potential. As such, to get a representative picture for the capabilities of our deep learning method, data from two large databases are used for both MOFs-CO_2 and COFs-CH_4 pairs. In this work, no molecular simulations were performed to generate the labels (adsorption uptakes) of the materials, since the datasets were already labeled. Information regarding the molecular simulations, can be found in the original works[2,3].

MOFs

With regards to CO_2, the evaluation of the proposed approach and its comparison with the conventional scheme where geometric descriptors are used, takes place on a subset of the University of Ottawa (UO) database[2]. The latter contains 8 geometric

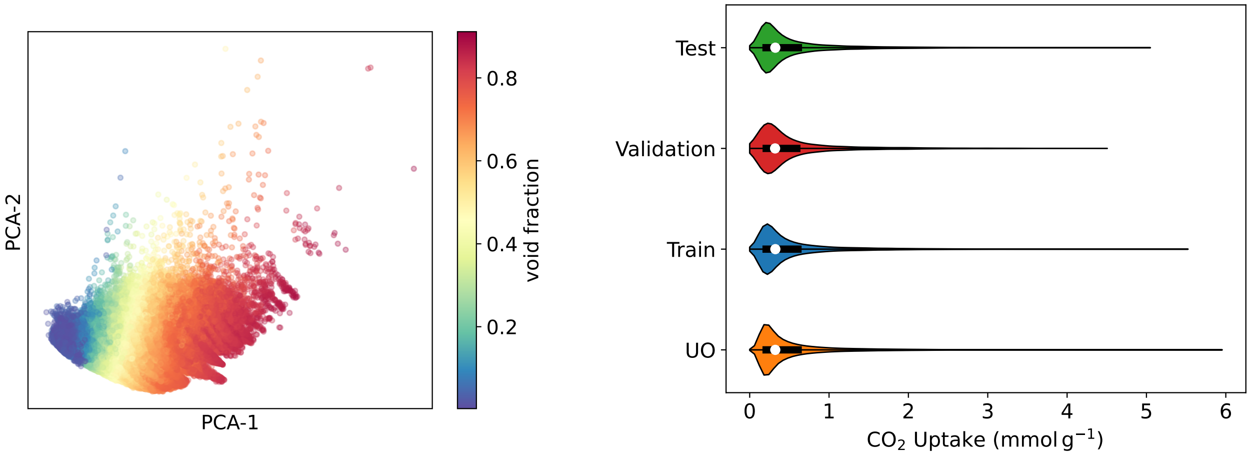

Figure 1. Visualizing the UO dataset. (Left) Dimensionality reduction of the MOFs dataset via PCA. Each point represents a material and is colorized by its void fraction. (Right) Violin plots for the CO_2 uptake. The left and the right end of the black bar represent the first and the third quartile, respectively. White dot denotes the median.

descriptors and CO_2 uptakes at different thermodynamic conditions for 324426 hypothetical MOFs. The geometric descriptors are: unit cell's mass and volume, gravimetric surface area, void fraction, void volume, largest free sphere diameter, largest included sphere along free sphere path diameter and largest included sphere diameter. In this work, the absolute uptake at 298 K and 0.15 bar is examined. A subset of 32432 MOFs serves as a training set, 5000 as a validation set (only for the CNN to select the number of epochs, see "Preprocessing and training details" subsection) and 27438 as a test set.

Please note that the training set size was varied (see "Learning Curves" section) for producing the learning curves shown in the manuscript. As such, the value 32432 is the maximum training set size for the MOFs-CO_2 case.

COFs

The transferability of the approach is evaluated on the COFs database created by Mercado et al[3], containing 69839 structures. This database provides data for 5 geometric descriptors and CH_4 uptakes at different thermodynamic conditions. The geometric descriptors are: mass density, gravimetric surface area, void fraction, pore limiting diameter and largest cavity diameter. In this work, CH_4 uptake at 298 K and 5.8 bar is examined. A subset of 55871 COFs form the training set and the remaining 13968 constitute the test set.

Please note that the training set size was varied (see "Learning Curves" section) for producing the learning curves shown in the manuscript. As such, the value 55871 is the maximum training set size for the COFs-CH_4 case.

Machine Learning Details

In all cases the geometric descriptors are coupled with the Random Forest algorithm (RF) as implemented in the `scikit-learn`[4] package (version 1.2.2). The `PyTorch`[5] framework (version 2.0.1cu+118) was used throughout this work for building and training the CNN models. The architecture of the CNN used in the present study, is summarized in Table 1.

The performance of all models in this work is measured by the coefficient of determination, R^2. Given a test set of size N, the latter is defined as:

$$R^2 = 1 - \frac{\sum_{i=1}^{N}(y_i - \hat{y}_i)^2}{\sum_{i=1}^{N}(y_i - \bar{y})^2} \qquad \text{or} \qquad R^2 = 1 - \frac{\frac{\sum_{i=1}^{N}(y_i-\hat{y}_i)^2}{N}}{\frac{\sum_{i=1}^{N}(y_i-\bar{y})^2}{N}} \qquad \text{or} \qquad R^2 = 1 - \frac{\text{MSE}^*}{\text{MSE}'} \tag{3}$$

where y_i, $\hat{y}_i$ are the ground truth (reference) value and predicted value of the i-th sample, respectively, and $\bar{y} = \sum_{i=1}^{N} y_i$, i.e. the mean value of y_i in the test set.

The R^2 metric compares the mean squared error of a model of interest MSE* (the numerator in Equation 3) with the mean squared error of a baseline model MSE$'$ (the denominator in Equation 3), which always predicts the mean value of the test set $\bar{y}$.

- When $R^2 < 0$, i.e. when numerator is greater than the denominator, the model of interest performs worse than the baseline model.

- When $R^2 = 0$, i.e. when numerator is equal to the denominator, the model of interest performs as good as the baseline model.
- When $R^2 > 0$, i.e. when numerator is smaller than the denominator, the model of interest performs better than the baseline model.

The higher the R^2 value the better the model. For a perfect model, i.e. one where $\hat{y}_i = y_i$ for each sample point, its mean squared error (the numerator) is 0 and as such, $R^2 = 1$.

In all cases where 95% confidence intervals (CI) are presented, they were calculated with the percentile bootstrap method[6] using 10000 bootstrapped samples from the corresponding test set.

Preprocessing and CNN training details

Preprocessing

The voxels of each material are standardized "on the fly" based on the training set statistics, prior to entering the CNN (this preprocessing step is applied both during training and inference). That is, the x_{ijk} voxel of a material enters the CNN as x'_{ijk}:

$$x'_{ijk} = \frac{x_{ijk} - \mu}{\sigma} \tag{4}$$

where μ, σ are the training mean and standard deviation, respectively, obtained as following:

$$\mu = \frac{1}{15625 \cdot N_{\text{train}}} \sum_{n=1}^{N_{\text{train}}} \sum_{i=1}^{25} \sum_{j=1}^{25} \sum_{k=1}^{25} x^n_{ijk} \quad \text{and} \quad \sigma = \sqrt{\frac{1}{(15625 \cdot N_{\text{train}}) - 1} \sum_{n=1}^{N_{\text{train}}} \sum_{i=1}^{25} \sum_{j=1}^{25} \sum_{k=1}^{25} (x^n_{ijk} - \mu)^2} \tag{5}$$

The upper limit 25 in the above sums is the value of grid size used in this work, 15625 is the total number of voxels in a voxelized PES (i.e. $25 \times 25 \times 25 = 15625$), while x^n_{ijk} is the ijk-th voxel of the n-th material in the training set. In other words, standardization is applied channel-wise (the voxelized PES is just a single-channel 3D image).

CNN training

Regarding CNN training, mean squared error is selected as loss function, weights are initialized according to the He scheme[7] and the Adam[8] optimizer is employed, with learning rate $\eta = 0.001$, $\beta_1 = 0.9$, $\beta_2 = 0.999$ and $\varepsilon = 1 \times 10^{-8}$. The batch size is set to 64, the CNN is trained for 50 epochs with data augmentation (see "Data augmentation" subsection) and the learning rate is being decayed by a factor of 0.5 every 10 epochs (learning rate scheduling).

The number of epochs was determined by examining the performance of CNN in the MOFs dataset. The CNN was trained with the largest training set (32432 samples) for a different number of epochs, namely 10, 20 and 50, and the value 50 was selected, since it showed the greatest performance (as measured by R^2) on the validation set (5000 samples). This value was used for all CNN models trained in this work. In other words, all CNN models are trained with the following setup:

1. **Architecture**: `RetNet`
   - Weight initialization: He initialization
2. **Loss function**: mean squared error

   $$\mathcal{L} = \frac{1}{|\mathcal{B}|} \sum_{i \in \mathcal{B}} (y_i - \hat{y}_i)^2$$

3. **Optimizer**: Adam
   - Batch $\mathcal{B} \subset \mathcal{D}_{\text{train}}$, sampled randomly without replacement from the training set $\mathcal{D}_{\text{train}}$
   - Batch size: $|\mathcal{B}| = 64$
   - Shuffle $\mathcal{D}_{\text{train}}$ after each epoch: `True`
   - Learning rate: $\eta = 0.001$

- Momentum decay: $\beta_1 = 0.9$
- Scaling decay: $\beta_2 = 0.999$
- Epsilon constant: $\varepsilon = 1 \times 10^{-8}$
- Number of epochs: 50
- Learning rate scheduling: decay η by 0.5 every 10 epochs

4. **Data augmentation**: geometric transformations with equal probability (see "Data augmentation" subsection)

   - Identity
   - Rotate90
   - Reflect
   - Flip

The training of CNN with the largest training set sizes took around 35 min and 45 min for MOFs-CO_2 (32432 training samples) and COFs-CH_4 (55871 training samples), respectively, on NVIDIA GeForce GTX 1650 Super (4 GB VRAM).

Layer	In	Out	Kernel size	Stride	Padding	Padding mode
Input						
Conv3d	1	12	3	1	1	"circular"
BatchNorm3d(`num_features=12`)						
LeakyReLU(`negative_slope=0.01`)						
Conv3d	12	24	3	1	0	
BatchNorm3d(`num_features=24`)						
LeakyReLU(`negative_slope=0.01`)						
MaxPool3d			2	2		
Conv3d	24	32	2	1	0	
BatchNorm3d(`num_features=32`)						
LeakyReLU(`negative_slope=0.01`)						
MaxPool3d			2	2		
Conv3d	32	64	2	1	0	
BatchNorm3d(`num_features=64`)						
LeakyReLU(`negative_slope=0.01`)						
Conv3d	64	120	2	1	0	
BatchNorm3d(`num_features=120`)						
LeakyReLU(`negative_slope=0.01`)						
Flatten						
Dropout(`p=0.3`)						
Linear	3240	84				
BatchNorm1d(`num_features=84`)						
LeakyReLU(`negative_slope=0.01`)						
Linear	84	20				
BatchNorm1d(`num_features=20`)						
LeakyReLU(`negative_slope=0.01`)						
Linear (Output)	20	1				

Table 1. `RetNet` architecture. An empty cell indicates that a parameter doesn't apply for the corresponding layer. For Conv3d layers, "In" and "Out" denote the number of input and output channels (aka feature maps), respectively. For Linear layers, "In" and "Out" denote the number of input and output features, respectively. For the Dropout layer[9], the parameter `p` denotes the dropout rate. In all Conv layers, the `bias=False` option was used.

Data augmentation

With this technique, the size of the training set is artificially increased, by applying geometric transformations on the voxelized PES that leave the label unchanged. Data augmentation helps the CNN to avoid memorization (e.g. specific orientations of the voxelized PES) and focus on the underlying patterns.

During each training iteration, the samples in the batch undergo the geometric transformations depicted in Figure 2. Only one of the transformations (including the identity one) is selected and applied randomly at each iteration, with equal probability. For example, at one iteration 90° rotation around the z-axis might be applied, while at another iteration flip along the x-axis is performed.

Figure 3 illustrates the performance difference when the CNN is trained on the MOFs dataset with and without data augmentation, for training set sizes $\{5000, 10000, 15000, 20000, 32432\}$.

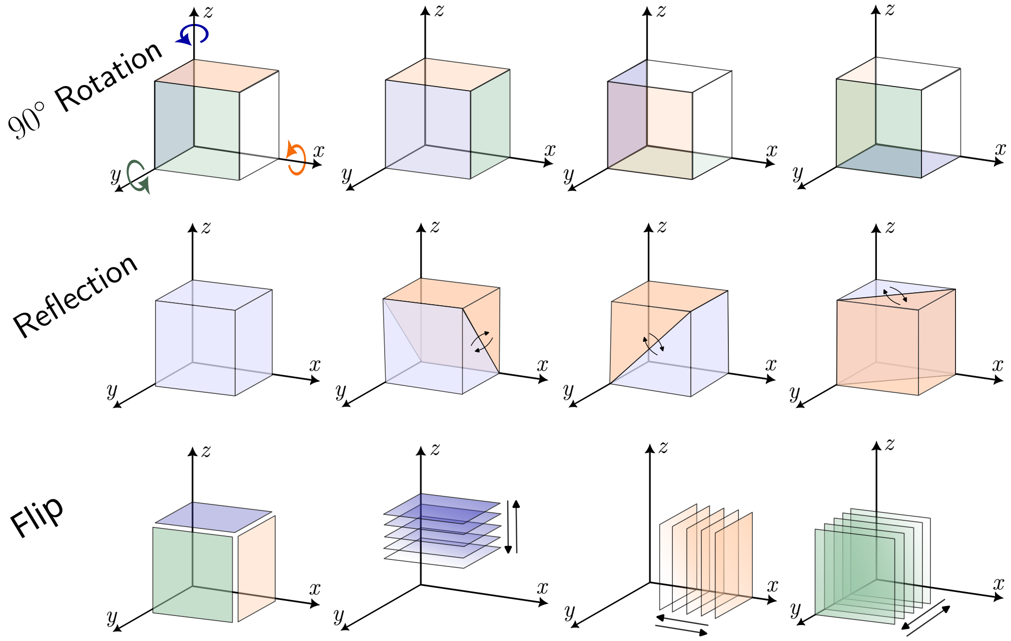

Figure 2. Geometric transformations for data augmentation. Rotation is performed either clockwise or counterclockwise around one of the three axes. The voxels can also be viewed as a stack of 2D slices along one of the three axes. In this view, reflection corresponds to transposing each slice while flip reverses the order of the slices. Reflection is performed along one of the zy, xz and xy planes while flip is performed along one of the three axes.

Learning curves

The learning curves shown in the manuscript were produced by varying the training set size and training the RF and CNN algorithms from scratch. The training set sizes examined were:

- MOFs-CO_2: $\{100, 500, 1000, 2000, 5000, 10000, 15000, 20000, 32432\}$
- COFs-CH_4: $\{5000, 10000, 15000, 20000, 35000, 55871\}$

In all cases, the resulting RF and CNN models were evaluated on the corresponding test sets, that is 27438 test samples for MOFs-CO_2 and 13968 test samples for COFs-CH_4.

Parity plots and fingerprints extracted from CNN

References

1. Rappe, A. K., Casewit, C. J., Colwell, K. S., Goddard, W. A. & Skiff, W. M. UFF, a full periodic table force field for molecular mechanics and molecular dynamics simulations. *J. Am. Chem. Soc.* **114**, 10024–10035, DOI: 10.1021/ja00051a040 (1992).

2. Boyd, P. G. *et al.* Data-driven design of metal-organic frameworks for wet flue gas CO_2 capture. *Nature* **576**, 253–256, DOI: 10.1038/s41586-019-1798-7 (2019).

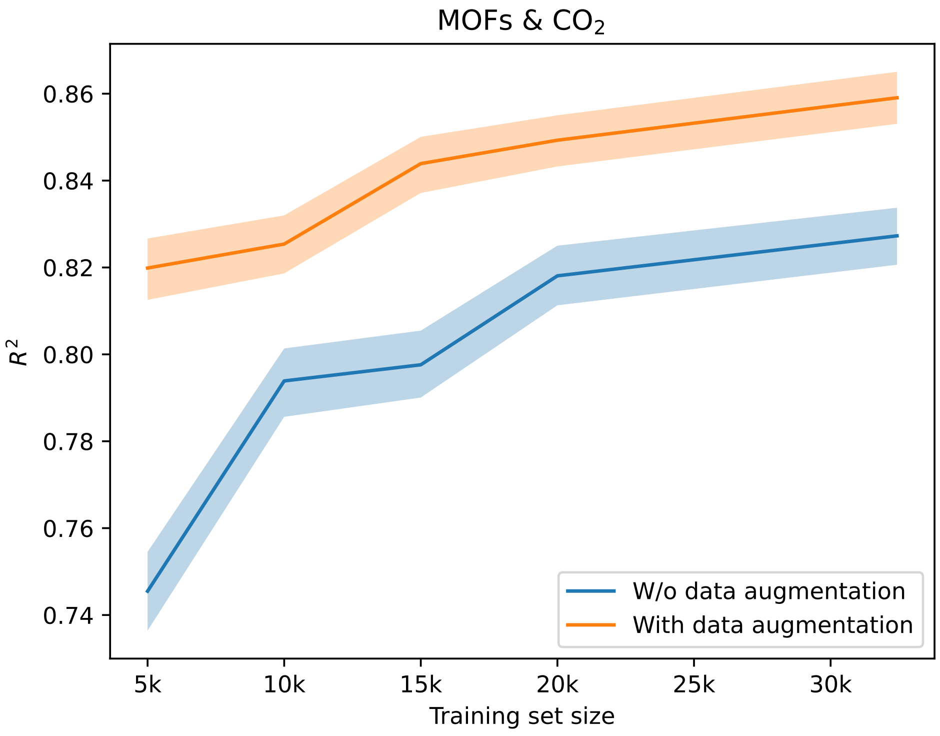

Figure 3. CNN performance on the test set (measured by R^2) as function of the training set size. Shaded areas correspond to the 95% CI.

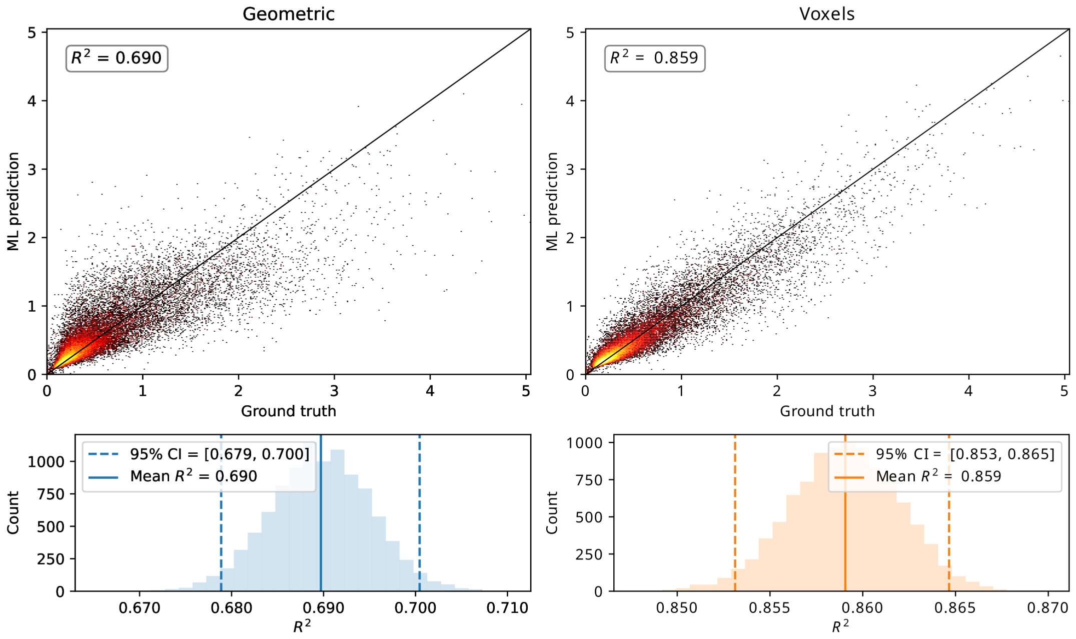

Figure 4. Parity plots (top) for the ML models with geometric descriptors (RF model) and energy voxels (CNN model) regarding CO_2 uptake in MOFs. Histograms (bottom) of the R^2 values used to construct the 95% CI. Both models were trained with the largest training set (32432 training samples). "Geometric" stands for geometric descriptors while "Voxels" stands for energy voxels.

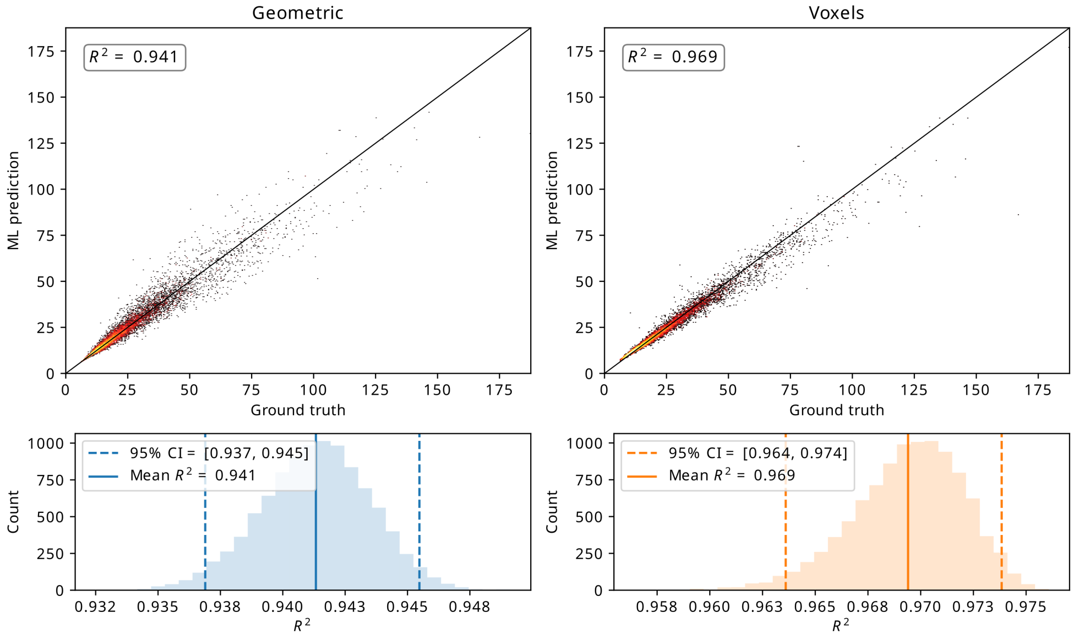

Figure 5. Parity plots (top) for the ML models with geometric descriptors (RF model) and energy voxels (CNN model) regarding CH_4 uptake in COFs. Histograms (bottom) of the R^2 values used to construct the 95% CI. Both models were trained with the largest training set (55871 training samples). “Geometric” stands for geometric descriptors while “Voxels” stands for energy voxels.

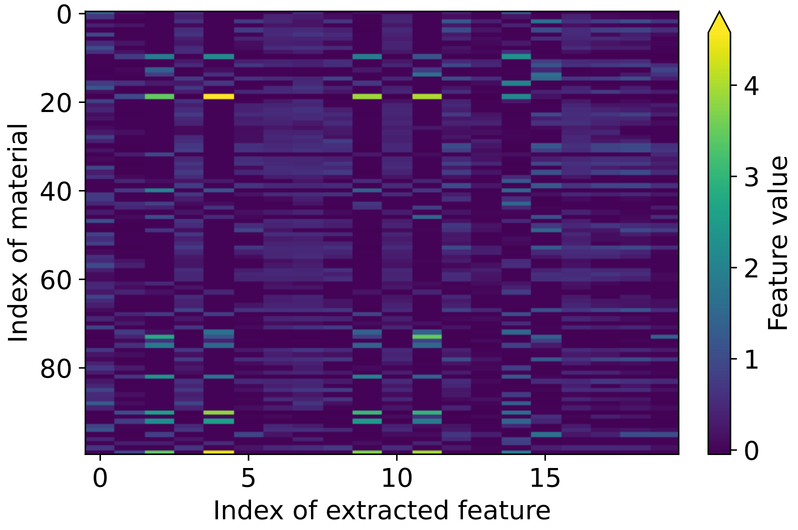

Figure 6. Output of the last LeakyReLU layer (i.e. extracted features/fingerpints) of `RetNet` trained on MOFs with the maximum training set size. The fingerprints for the first 100 materials of the training set are depicted.

3. Mercado, R. *et al.* In silico design of 2d and 3d covalent organic frameworks for methane storage applications. *Chem. Mater.* **30**, 5069–5086, DOI: 10.1021/acs.chemmater.8b01425 (2018).

4. Pedregosa, F. *et al.* Scikit-learn: Machine learning in Python. *J. Mach. Learn. Res.* **12**, 2825–2830 (2011).

5. Paszke, A. *et al.* Pytorch: An imperative style, high-performance deep learning library. In *Advances in Neural Information Processing Systems 32*, 8024–8035 (Curran Associates, Inc., 2019).

6. Efron, B. & Tibshirani, R. *An Introduction to the Bootstrap*. Chapman & Hall/CRC Monographs on Statistics & Applied Probability (Taylor & Francis, 1994).

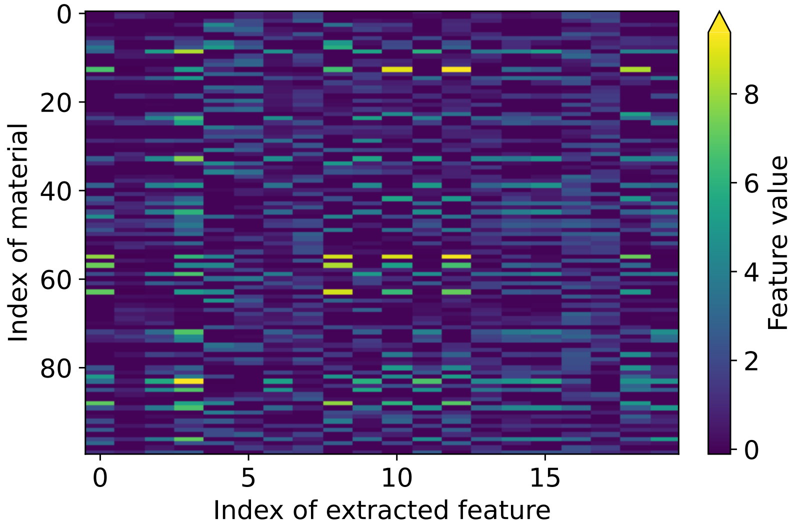

Figure 7. Output of the last LeakyReLU layer (i.e. extracted features/fingerpints) of `RetNet` trained on COFs with the maximum training set size. The fingerprints for the first 100 materials of the training set are depicted.

7. He, K., Zhang, X., Ren, S. & Sun, J. Delving deep into rectifiers: Surpassing human-level performance on imagenet classification (2015). 1502.01852.

8. Kingma, D. P. & Ba, J. Adam: A method for stochastic optimization (2017). 1412.6980.

9. Srivastava, N., Hinton, G., Krizhevsky, A., Sutskever, I. & Salakhutdinov, R. Dropout: A simple way to prevent neural networks from overfitting. *J. Mach. Learn. Res.* **15**, 1929–1958 (2014).
